# Supplementary material for: In situ characterization of vacancy ordering in Ge-Sb-Te phase-change memory alloys
Source: Fundam Res. 2022 Sep 30;4(5):1235–42. doi: 10.1016/j.fmre.2022.09.010 (PMC11489497; doi:10.1016/j.fmre.2022.09.010)
Supplement: Supplementary file 1 [file mmc1.docx]

**Supplementary Material**

**for**

***In situ characterization of vacancy ordering in Ge-Sb-Te phase-change memory alloys***

Ting-Ting Jiang^1#^, Xu-Dong Wang^1#^, Jiang-Jing Wang^1,2^, Han-Yi Zhang^1^, Lu Lu^3^, Chunlin Jia^3^, Matthias Wuttig^2,4,5^, Riccardo Mazzarello^6^, Wei Zhang^1^*, En Ma^1^

^1^Center for Alloy Innovation and Design (CAID), State Key Laboratory for Mechanical Behavior of Materials, Xi’an Jiaotong University, Xi'an, 710049, China.

^2^Institute of Physics IA, RWTH Aachen University, Aachen, 52074, Germany

^3^The School of Microelectronics, State Key Laboratory for Mechanical Behavior of Materials, Xi’an Jiaotong, University, Xi'an, 710049, China

^4^JARA-FIT and JARA-HPC, RWTH Aachen University, Aachen, 52056, Germany

^5^Peter Grünberg Institute (PGI 10), Forschungszentrum Jülich GmbH, Jülich, 52425, Germany

^6^Department of Physics, Sapienza University of Rome, Rome, 00185, Italy

^#^These authors contributed equally to this work.

*E-mail: [wzhang0@mail.xjtu.edu.cn](mailto:wzhang0@mail.xjtu.edu.cn)


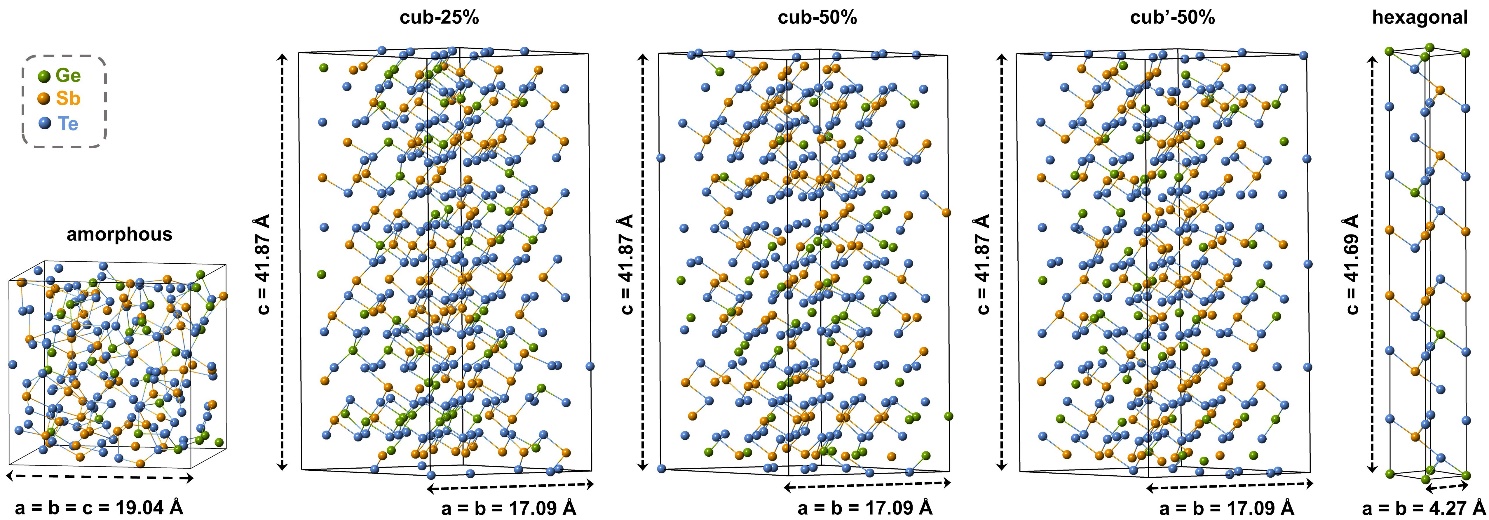


**Figure S1.** DFT relaxed structures for the amorphous, cub-25%, cub-50%, cub’-50% and hexagonal phase. Parts of the atomic structures are shown in Figure 5. The amorphous model contains 189 atoms. The cubic supercell models contain 336 atoms. The hexagonal unit cell model contains 21 atoms.


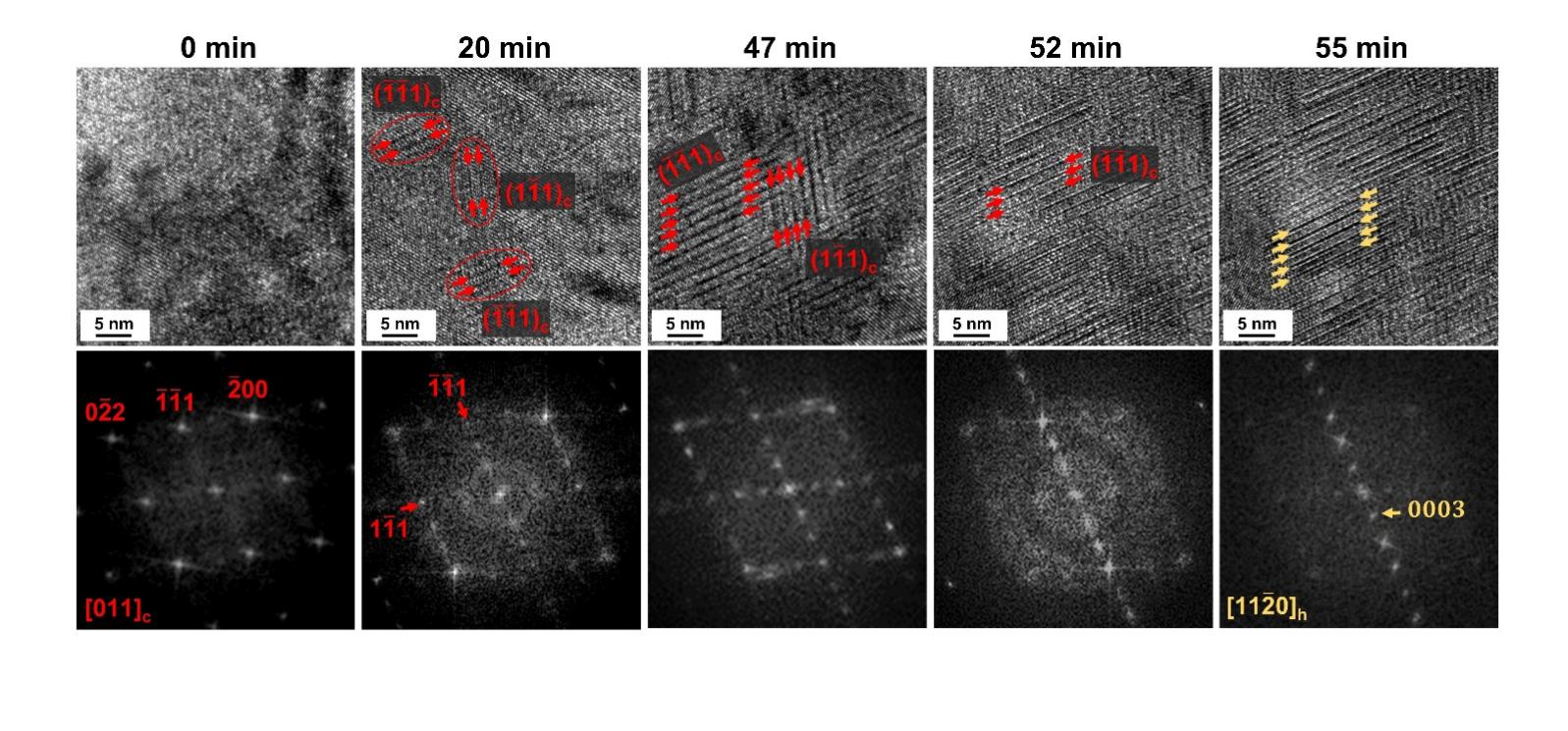


**Figure S2.** *In situ* observation of vacancy ordering in another local area extracted from Figure 1b. Vacancy ordering appears in the {111}_c_ planes of the cub-phase (red arrows) and in hex-phase lattices indicated by yellow arrows.


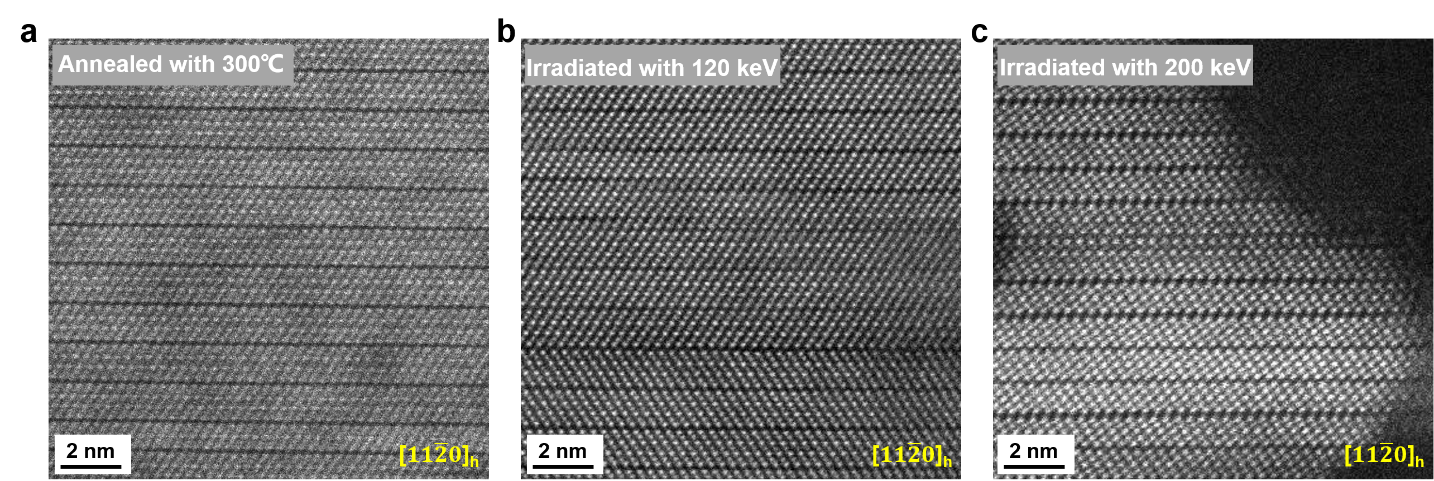


**Figure S3.** The HAADF images on (a) the thermally annealed sample at 300 ^o^C, (b) the irradiated sample performed on JEM-200 CX TEM operated at 120 keV, and (c) the irradiated sample performed on JEOL JEM-2100F TEM operated at 200 keV. The former two samples show SL blocks, while the latter shows QL blocks. The dark regions in (c) are in the amorphous phase with Ge-richer composition.

**
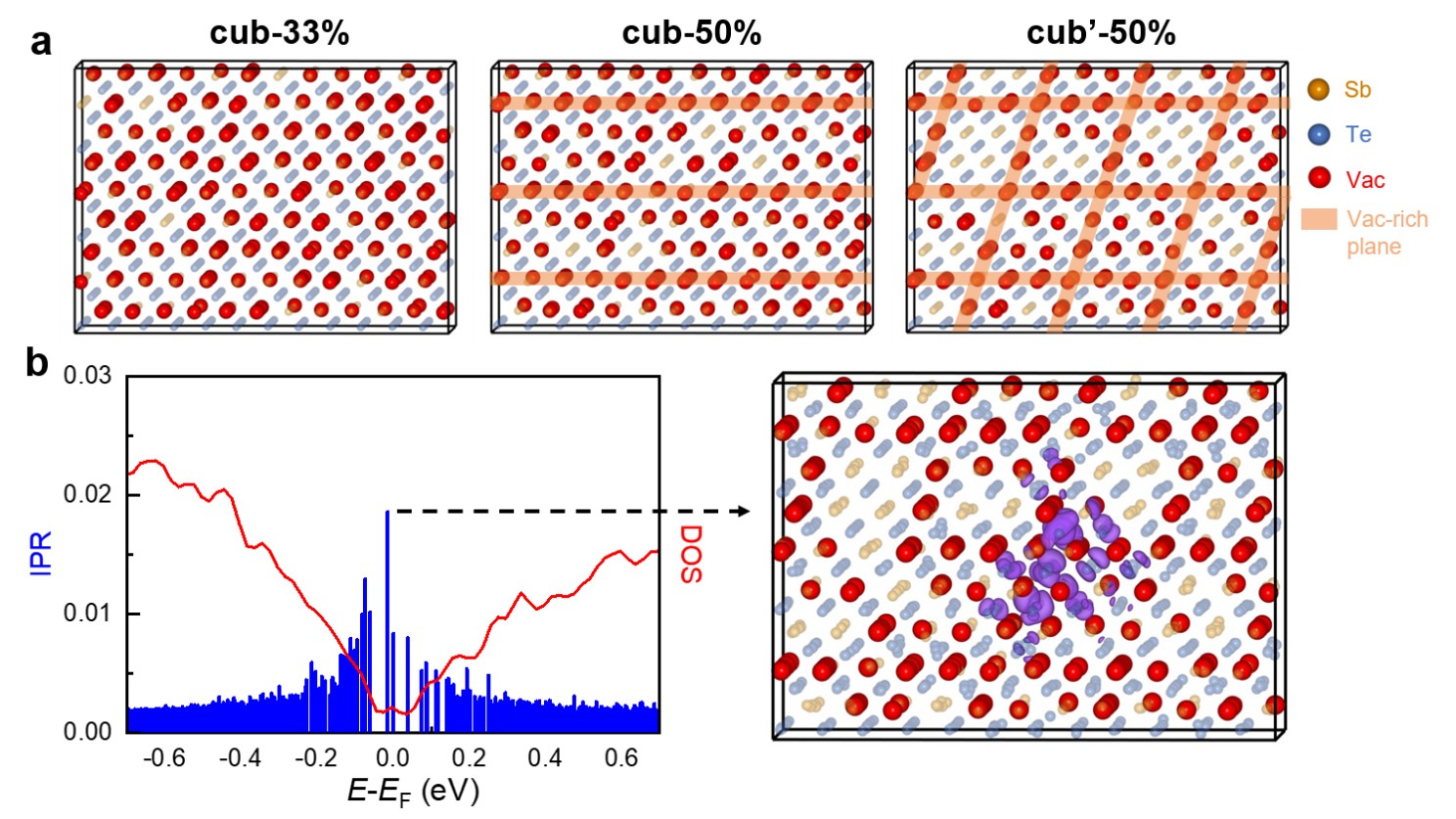
**

**Figure S4.** Atomic and electronic structure of Sb_2_Te_3_. (a) Atomic structures of cub-33%, cub-50% and cub’-50%, respectively. Each model contains 1080 atoms. The yellow, blue and red spheres represent Sb, Te and vacancy, respectively. The vacancy-rich planes are highlighted in orange. Five independent models were considered. The average total energy values show that cub-50% is ~5.6 meV/atom lower than cub-33%, while cub’-50% is slightly lower in energy than cub-50% by ~1.5 meV/atom. (b) The calculated DOS, IPR and charge isosurface of an occupied state (indicated by the dashed arrow) of the cub’-50% model. The isosurface rendered in purple corresponds to an isovalue of 0.012 a.u.
